# CA1 contributes to microcalcification and tumourigenesis in breast cancer

**DOI:** 10.1186/s12885-015-1707-x

**Published:** 2015-10-12

**Authors:** Yabing Zheng, Bing Xu, Yan Zhao, He Gu, Chang Li, Yao Wang, Xiaotian Chang

**Affiliations:** 1 Medical Research Center of Shandong Provincial Qianfoshan Hospital, Shandong University, Jingshi Road 16766, Jinan, Shandong 250014 P. R. China; 2 People’s Central Hospital of Tengzhou, Tengzhou, Shandong 277500 P. R. China

**Keywords:** Breast cancer, Microcalcification, Tumourigenesis, Carbonic anhydrase I (CA1), Androgen receptor (AR), X-box binding protein 1 (XBP1)

## Abstract

**Background:**

Although mammary microcalcification is frequently observed and has been associated with poor survival in patients with breast cancer, the genesis of calcification remains unclear. Carbonic anhydrase I (CA1) has been shown to promote calcification by catalysing the hydration of CO_2_. This study aimed to determine whether CA1 was correlated with microcalcification and with other processes that are involved in breast cancer tumourigenesis.

**Methods:**

CA1 expression in breast cancer tissues and blood samples was detected using western blotting, real-time PCR, immunohistochemistry and ELISA. Calcification was induced in the cultured 4T1 cell line originating from mouse breast tumours, using ascorbic acid and β-glycerophosphate. Acetazolamide, a chemical inhibitor of CA1, was also added to the culture to determine the role of CA1 in calcification. The MCF-7 human breast cancer cell line was treated with anti-CA1 siRNA and was assessed using a CCK-8 cell proliferation assay, an annexin V cell apoptosis assay, transwell migration assay and a human breast cancer PCR array. The tag SNP rs725605, which is located in the CA1 locus, was genotyped using TaqMan® genotyping.

**Results:**

Increased CA1 expression was detected in samples of breast carcinoma tissues and blood obtained from patients with breast cancer. A total of 15.3 % of these blood samples exhibited a 2.1-fold or higher level of CA1 expression, compared to the average level of CA1 expression in samples from healthy controls. Following the induction of calcification of 4T1 cells, both the number of calcium-rich deposits and the expression of CA1 increased, whereas the calcification and CA1 expression were significantly supressed in the presence of acetazolamide. Increased migration and apoptosis were observed in MCF-7 cells that were treated with anti-CA1 siRNA. The PCR array detected up-regulation of the androgen receptor (AR) and down-regulation of X-box binding protein 1 (XBP1) in the treated MCF-7 cells. Significant differences in the allele and genotype frequencies of rs725605 were detected in the cohort of patients with breast cancer but not in other tumours.

**Conclusion:**

The results of this study suggested that CA1 is a potential oncogene and that it contributes to abnormal cell calcification, apoptosis and migration in breast cancer.

**Electronic supplementary material:**

The online version of this article (doi:10.1186/s12885-015-1707-x) contains supplementary material, which is available to authorized users.

## Background

Mammary microcalcification is frequently associated with poor survival, and it occurs in 30 to 50 % of breast cancer patients [1]. Although the diagnostic value of microcalcification in patients with breast cancer is of great importance, the genesis of this calcification remains unclear. There are two distinct forms of mammary calcifications: calcium oxalate and hydroxyapatite [2, 3]. Hydroxyapatite is also a well-documented component of bone, and the deposition of it in bone tissue requires the coordinated expression of several bone matrix proteins, which are synthesised by cells of osteoblastic lineage [4]. There has been evidence indicating that calcium carbonate is involved in initial bone formation, although mineral deposits are typically comprised of calcium phosphate and not calcium carbonate [5–7]. CA1 (carbonic anhydrase 1) is a member of the carbonic anhydrase (CA) family, and it catalyses the reversible hydration and dehydration reactions of CO_2_/H_2_CO_3_ [8]. *In vitro* assays have demonstrated that CA1 not only enhances hydration reactions but also promotes the formation of CaCO_3_ [9, 10]. In a recent study, we detected increased expression of CA1 in Saos-2, a human osteosarcoma cell line, following the induction of calcification with ascorbic acid and β-glycerophosphate. Following treatment with acetazolamide, an anti-CA1 drug, both CA1 expression and the formation of mineralised nodules decreased [11]. Furthermore, overexpression of CA1 was detected in the synovial tissues of patients with ankylosing spondylitis, a disease characterised by abnormal bone formation in the spinal and sacroiliac joints [12]. The gene that encodes CA1 is susceptible to ankylosing spondylitis [11], and transgenic mice with overexpression of CA1 exhibited bone fusion in their paws and spines [13]. These results suggest that CA1 might play essential roles in bio-mineralisation and new bone formation.

In the present study, we hypothesised that the up-regulation of CA1 in breast tumours stimulates calcium precipitation, similar to what occurs in bone tissue. To test this hypothesis, calcification was induced in 4T1 cells, which originated from a murine mammary adenocarcinoma. Cox et al. recently reported a novel *in vitro* model of mammary mineralisation using 4T1 cells [14, 15]. Using this model, we investigated the involvement of CA1 in bio-mineralisation and calcification. Additionally, the effects of CA1 on cell proliferation and cell apoptosis were investigated in anti-CA1 siRNA-treated MCF-7 cells that originated from human breast cancer, and the pathogenic pathway was analysed using a PCR array that contained breast cancer genes. We also used a Taqman genotyping method to determine the correlations between common polymorphisms in the CA1 encoding gene and breast cancer. In a previous study, we genotyped 13 different tag SNPs in the CA1 gene to determine whether a potential association existed between the CA1 gene and ankylosing spondylitis, using a custom-designed Illumina 96-SNP VeraCode microarray (Illumina). We subsequently performed Taqman genotyping of the tag SNPs that exhibited significant associations with the disease in a large cohort of the patients. Both microarray and Taqman genotyping demonstrated that the allele and gene frequencies of rs725605 were statistically significantly associated with patients who had ankylosing spondylitis [11]. At that time, we tested five SNPs by Taqman genotyping, and only rs725605 produced favourable results. Thus, we continued to genotype this tag SNP to determine whether the CA1 gene had possible associations with the various types of tumours that were examined in the current study. Furthermore, we examined the CA1 expression in breast tumour tissues and blood samples from patients.

## Methods

### Tissue collection

In the present study, all of the solid tissue samples and blood samples that were used were collected from patients of Shandong Provincial Qianfoshan Hospital (Jinan, China). The tumour diagnoses were verified using histological methods, and pathological categorisations were performed according to the World Health Organisation (WHO) classification system. All of the included patients signed informed consent forms, and this study was approved by the ethics committee of Shandong Provincial Qianfoshan Hospital (reference number 2013012). All research involving human subjects (including human material or human data) that is reported in the manuscript was conducted in compliance with the Helsinki Declaration.

### SNP selection and Taqman genotyping

The tag SNP rs725605 was identified by searching the HapMap database. This tag SNP was selected on the basis of linkage disequilibrium patterns that were observed in samples obtained from the Han Chinese population of Beijing, who were genotyped as part of the International HapMap Project. Illumina microarray and Taqman genotyping assays confirmed the location of tag SNP rs725605 in the CA1 locus [11].

To determine the potential associations between this tag SNP and various cancer risks, genotyping was performed using TaqMan® technology in cohorts of patients who had breast cancer (*n* = 285, 285 women, mean age = 47.65 years old), colon cancer (*n* = 145, 55 women, mean age = 54.13 years old), oesophageal cancer (*n* = 285, 40 women, mean age = 61.20 years old), cervical cancer (*n* = 190, 190 women, mean age = 52.75 years old), liver cancer (*n* = 190, 42 women, mean age = 54.05 years old), lung cancer (*n* = 190, 56 women, mean age = 58.17 years old), gastric cancer (*n* = 285, 71 women, mean age = 56.83 years old) or rectal cancer (*n* = 137, 50 women, mean age = 54.61 years old), as well as in healthy controls (*n* = 285, 71 women, mean age = 38.42 years old). Blood samples were collected and stored in Monovette tubes containing 3.8 % sodium citrate.

Genomic DNA was extracted from whole blood samples with an Omega E-Z 96 Blood DNA kit (Omega, USA) according to the manufacturer’s protocol. Genotyping assays were run on a ViiA 7 DX (Life Technology) and were evaluated according to the manufacturer’s instructions. Each reaction was performed in a total volume of 10 μl using the following amplification protocol: denaturation at 95 °C for 10 min, followed by 50 cycles of denaturation at 95 °C for 15 s and finishing with annealing and extension at 60 °C for 1 min. The genotype of each sample was determined by measuring allele-specific fluorescence using Taqman Genotyper software, version 1.2 (Life Technology). Duplicate samples and negative controls were included to verify the accuracy of the genotyping.

Genotyping quality was evaluated using a detailed quality control procedure that consisted of a >95 % successful call rate, duplicate calling of genotypes, internal positive control samples and Hardy-Weinberg Equilibrium (HWE) testing. SNPs were analysed for associations by comparing the MAFs (minor allele frequencies) between cases and controls. Dominant and recessive models were considered with respect to the minor allele. Associations of SNPs with diseases were evaluated using odds ratios (ORs) with 95 % confidence intervals (CIs). Fisher’s exact test was used for comparisons between categorical variables. p values less than 0.05 were considered statistically significant. Genotypic association was assessed using SHEsis software [16]. Multiple-test correction, including genomic-control correction and Bonferroni’s single-step correction, was performed using Plink software, version 1.07 (http://pngu.mgh.harvard.edu/purcell/plink/).

### Western blot analysis

Breast tissues were collected during galactophore operations from patients with breast cancer (*n* = 7, 7 women; 34–64 years old, mean 52) or breast fibroadenoma (*n* = 7, 7 female subjects; 17–59 years old, mean 35). Two hundred micrograms of each of the breast cancer tumour and breast fibroadenoma samples were individually homogenised in Cell Lysis Solution (Sigma) and were centrifuged at 12,000 × g for 30 min at 4 °C. The supernatants were collected after centrifugation, and protein concentrations were determined using a BCA protein assay kit (Pierce). In total, 30 μg of protein were loaded and separated by sodium dodecyl sulphate-polyacrylamide gel electrophoresis (SDS-PAGE), transferred onto a polyvinylidene membrane and probed with an anti-human carbonic anhydrase 1 antibody (1:1000; Abcam). The antibody was prepared by immunising a goat with carbonic anhydrase I that was extracted from human erythrocytes. The manufacturer confirmed that no cross-reactivity existed with other carbonic anhydrases. The membranes were subsequently rinsed with washing solution and incubated with sheep anti-goat IgG conjugated with peroxidase (1:500; Sigma-Aldrich) for 30 min. Following a washing step, the immunosignal was detected using an Enhanced Chemiluminescence (ECL) Plus kit (Millipore) and was quantified using ImageQuant software, version 5.2 (GE Healthcare). Another membrane was prepared using the same protocol and was probed with an anti-GADPH antibody (Santa Cruz) to normalise sample loading.

### Immunohistochemistry

Tissue array slides were obtained commercially from Alenabio (China). The array slides contained 40 different invasive breast ductal carcinoma and 8 different normal tissue samples. Clinical data, including the age, sex, clinical pathological diagnosis and origin of every participant, were provided by the manufacturer. Tissue sections were deparaffinised and rehydrated using standard procedures. Prior to the application of the antibodies, the tissue sections were heated at 95 °C for 10 min in citrate buffer solution (Sigma) to facilitate antigen recovery, followed by incubation with an endogenous peroxidase inhibitor (MaixinBio, China) for 30 min at room temperature. After washing with PBS buffer, the section was incubated with anti-CA1 overnight at 4 °C. The CA1 antibody was prepared as described above. The immunoreaction was processed using the UltraSensitive TM S-P Kit (Maixin-Bio, China) according to the manufacturer’s instructions. The immune-reactive signal was visualised using DAB substrate, which stains target proteins yellow. Cell structures were counterstained with haematoxylin.

### Real-time PCR

Breast tissues were collected during galactophore operations that were performed on patients with breast cancer (*n* = 12, 12 women; 31–68 years old, mean 51) or breast fibroadenoma (*n* = 12, 12 women; 12–59 years old, mean 33). Total RNA extracts from breast cancer and breast fibroadenoma tissues were reverse-transcribed using an RNA PCR Kit (TaKaRa). Real-time PCR was conducted using a ViiA 7 DX. The relative levels of mRNA expression were calculated using a comparative threshold cycle (Ct) method. The relative expression levels of the target gene were normalised to the relative levels of GAPDH mRNA. The primer sequences that were used for the amplification of CA1 and GAPDH are described in Table 1. The reaction conditions were as follows: 10 s at 65 °C, followed by 40 cycles of 5 s at 60 °C and 10 s at 72 °C and then 30 s at 65 °C. The experiment was performed in triplicate, and the PCR products were confirmed using a melting curve analysis. Additionally, real-time PCR was used to verify the PCR array results. The primer sequences that were used for the amplification of AR (androgen receptor), GSTP1 (glutathione S-transferase pi 1), PTGS2 (prostaglandin-endoperoxide synthase 2 [prostaglandin G/H synthase and cyclooxygenase]), SNAI2 (snail family zinc finger 2) and XBP1 (X-box binding protein 1) are also shown in Table 1.

**Table 1:** Prime sequences for real time PCR (5'- > 3')

	Forward primer	Reverse primer
CA1	GCTACAGGCTCTTTCAGTT	GACTCCATCCACTGTATGTT
GAPDH	ACCACAGTCCATGCCATCAC	TCCACCACCCTGTTGCTGTA
AR	CCAGGGACCATGTTTTGCC	CGAAGACGACAAGATGGACAA
GSTP1	CCCTACACCGTGGTCTATTTCC	CAGGAGGCTTTGAGTGAGC
PTGS2	TAAGTGCGATTGTACCCGGAC	TTTGTAGCCATAGTCAGCATTGT
SNAI2	TGTGACAAGGAATATGTGAGCC	TGAGCCCTCAGATTTGACCTG
XBP1	CCCTCCAGAACATCTCCCCAT	ACATGACTGGGTCCAAGTTGT

### ELISA

Blood samples were collected from patients with breast cancer (*n* = 92, 90 women; 22–78 years old, mean 53) and healthy volunteers (*n* = 84, 82 women; 23–77 years old, mean 52). The blood samples were centrifuged at 3000 × g for 10 min at 4 °C to remove debris. The samples were diluted 5-fold with 0.05 M carbonate-bicarbonate buffer (pH 9.6) and were used to coat 96-well ELISA microplates (Costar), followed by overnight incubation at 4 °C. After a brief washing with PBS containing 0.1 % Tween-20 (PBST), the plates were blocked with 5 % non-fat dry milk for 1 h at room temperature. The anti-CA1 antibody was diluted 1000-fold with PBST and was added to the plate, and the plate was then incubated for 2 h at room temperature. After washing with PBST, a 1000-fold dilution of HRP-conjugated anti-goat total IgG (Sigma) was added, and the plate was incubated at room temperature for 1 h. The plates were washed three times and developed using tetramethyl benzidine (TMB, Sigma) as a substrate. O.D. was measured with a microplate reader at 450 nm.

### Cell culture and induction of bio-mineralisation

The 4T1 cell line, which originated from a murine mammary adenocarcinoma, was maintained in regular growth media that consisted of low glucose DMEM, 10 % FBS and 1 % penicillin/streptomycin. The cell line was purchased from the American Tissue Culture Collection. All of the cell culture reagents were purchased from Sigma-Aldrich. The 4T1 cells were seeded into 6-well culture plates at a density of 10^5^ cells/well and were induced for calcification with an osteogenic cocktail (OC) (50 g/ml ascorbic acid, 10 mM β-glycerophosphate) the following day. The cells were maintained in an atmosphere of 37 °C and 5 % CO_2_, and half of the medium was replenished every 3 days until the 28th day. This protocol design was based on a study by Cox et al. [14, 15]. 

To verify the effects of CA1 expression on bio-mineralisation, acetazolamide, a chemical inhibitor of CA1, was used to treat the 4T1 cells. Acetazolamide (Sigma) was dissolved in dimethyl sulphoxide (DMSO, Solabio) at a concentration of 1 % and was added to cell cultures that were grown to 70 % confluence in OC. The cultures were grown in the presence of acetazolamide at a final concentration of 1 mM. The 4T1 cells were also cultured in OC with 1 % DMSO as a control. The protocol was based on a study by Hall et al. [17, 18].

### Alizarin Red-S staining and quantification of bio-mineralisation

The cultured cells were washed with phosphate-buffered saline (PBS, Solabio) and were stained with 0.5 % (*w/v*) Alizarin Red-S (AR-S, Solabio) in PBS (pH = 5.0) for 30 min at room temperature. After being washed four times with PBS, the stained cells were photographed. The cultured cells were then de-stained with 10 % (*w/v*) cetylpyridinium chloride (Biobasic) in PBS (pH = 7.0) for 1 h at room temperature. The AR-S concentration was determined by measuring absorbance at 562 nm. The protocol was based on previously published studies [11, 19].

Expression levels of bone sialoprotein (BSP) and osteocalcin (OCN), two bone matrix proteins, were determined using real-time PCR. The expression of CA1 in the treated 4T1 cells was determined using real-time PCR and western blot analysis.

### Inhibiting CA1 expression with siRNAs in MCF-7 cells

The MCF-7 cell line, which originated from human breast cancer mammary epithelium, was cultured in regular growth media that consisted of RPMI-1640, 10 % FBS and 1 % penicillin/streptomycin. The cell line was purchased from the American Tissue Culture Collection. All of the cell culture reagents were purchased from Sigma-Aldrich. siRNA oligonucleotides targeting CA1 (target mRNA sequence: 5′-TCTACTCTCCTTCCTTCAT -3′) were designed and synthesised by RIBOBIO. The cultured MCF-7 cells were transfected with the siRNAs at 20 nM using HiPerFect transfection reagent (Qiagen), according to the manufacturer’s protocol. The cells were harvested for analysis 72 h after the transfection. Parallel experiments with AllStars siRNAs, which was provided with the kit, were used as a negative control. The AllStars siRNA has no homology to any known mammalian gene and therefore should not affect any gene expression. Additionally, the MCF-7 cells that were transfected with HiPerFect transfection reagent were also used as a control in the experiment.

### Cell proliferation assay

MCF-7 cells were seeded into 96-well culture plates and incubated until they reached 80 % confluence. The culture was then treated with anti-CA1 siRNA and incubated for 24–72 h at 37 °C in 5 % CO_2_. Following the addition of 10 μl of Cell Counting Kit-8 (CCK-8, Dojindo) solution to each well, the cells were incubated for an additional 4 h. Absorbance was measured at 450 nm with a spectrophotometer (Spectramax 190; Molecular Devices). Growth curves were generated from the average values of five wells per group.

The proliferation of the 4T1 cells that were cultured with OC was measured using the same protocol.

### Cell apoptosis assay

Apoptosis in the siRNA-treated MCF-7 cells was analysed using flow cytometry (FACSAria II, BD Biosciences). The cultured cells were washed twice with PBS and were resuspended in binding buffer at a concentration of 1 × 10^6^ cells/ml. The cell suspensions (1 × 10^5^ cells/100 μl) were transferred into 5 ml culture tubes, and 5 μl of annexin V-phycoerythrin (eBioscience) and 5 μl of 7-amino-actinomycin (eBioscience) were then added. The cells were gently vortexed and incubated at room temperature in the dark for 15 min. Subsequently, another 400 μl of aliquot of binding buffer were added. Flow cytometry was performed within 4 h of staining.

### Transwell migration assay

Transwell inserts (8.0 μm pore size) with a polycarbonate filter (Costar®) were used to examine the effects of CA1 on cell migration. siRNA-treated MCF-7 cells (5 × 10^4^ cells/200 μl) were suspended in serum-free media and added to the upper chamber, and 500 μl of complete DMEM media were added to the lower chamber. Following incubation for 24 h, the filter was immersed in methanol for 15 min at room temperature and then was treated with 0.25 % crystal violet stain for 10 min at room temperature prior to washing with water. The cells that had migrated to the lower side of the filter were counted with an inverted fluorescence microscope.

### PCR arrays and data verification

A Human Breast Cancer RT^2^ Profiler™ PCR Array (Qiagen) was used to screen for breast tumour genes that were affected by CA1 expression. The breast cancer pathway PCR array profiled the expression of 84 different key genes that are commonly involved in the dysregulation of signal transduction and other normal biological processes during breast carcinogenesis. PCR array analysis was conducted using a ViiA7 DX according to the manufacturer’s instructions. The procedure begins with the conversion of experimental RNA samples into first-strand cDNA using a RT^2^ First Strand Kit. Subsequently, the cDNA is mixed with an appropriate RT^2^ SYBR Green Mastermix. This mixture is then aliquoted into the wells of the RT^2^ Profiler PCR Array. Real-time PCR detection was performed under the following thermal cycling conditions: 95 °C for 10 min, 40 cycles of 95 °C for 15 s and 1 cycle of 60 °C for 1 min. Relative expressions were determined using the data that were generated with the real-time cycler and the ∆∆CT method. Another PCR array experiment was prepared with total RNA extracted from the cells with treatment of Allstars siRNA. The transcription levels of the target genes were normalised to their corresponding expression levels in the cells that were treated with Allstars siRNA. Data analysis was performed using Web-based RT^2^ Profiler PCR Array Data Analysis software (http://pcrdataanalysis.sabiosciences.com/pcr/arrayanalysis.php, SA Biosciences). Fold-changes in gene expression were calculated and expressed as log-normalised ratios of the siRNA treated cells/controls. According to the instructions of the manufacturer, genes with at least a 4-fold changes in expression were considered to be biologically significant in the study. The results of the PCR array assay were confirmed by performing real-time PCR on RNA extracts from anti-CA1 siRNA-treated MCF-7 cells.

### Statistical analyses

Statistical analyses of the data were performed using SPSS software, version 16 (SPSS). Multiple comparisons were conducted using ANOVA. The *t*-test was conducted to assess significant differences between two groups. P values less than 0.05 were considered significant. The data are presented as standard deviations.

## Results

### Genotyping rs725605 in cohorts of patients with tumours

Using the TaqMan method, genotyping of the tag SNP rs725605 was performed in samples obtained from cohorts of patients with breast cancer, colon cancer, oesophageal cancer, cervical cancer, liver cancer, lung cancer, gastric cancer, or rectal cancer, in addition to healthy controls. The allelic and gene frequencies of the SNP did not deviate from HWE in any of the cohorts. The allele frequency (OR = 0.722663, 95 % CI = 0.570361–0.915634, *p* = 0.007104) and gene frequency (*p* = 0.029009) of SNP rs725605 were both statistically associated with breast cancer. Following multivariate logistic regression analysis using Plink software, version 1.07, the correlations between the SNP frequencies and breast cancer were found to be statistically significant. The genotyping data did not indicate significant differences in the allelic or genotypic frequencies of rs725605 (*p* > 0.05) between patients with colon cancer, oesophageal cancer, cervical cancer, liver cancer, lung cancer, gastric cancer or rectal cancer and healthy controls. The results are shown in Table 2. SNP rs725605 is located in the intron 1 region of the gene that encodes CA1.

**Table 2:** Taqman genotyping (rs725605-CA1) result (control *n* = 288)

SNP identity-gene	Breast cancer *n* = 278		Rectal carcinoma *n* = 132		Colon carcinoma *n* = 140		Cervical carcinoma *n* = 179		Esophageal carcinoma *n* = 259		Gastric carcinoma *n* = 263		Liver cancer *n* = 186		Lung carcinoma *n* = 181	
Allele	A	G	A	G	A	G	A	G	A	G	A	G	A	G	A	G
Case (freq)	220 (0.396)		118 (0.447)		113 (0.404)		163 (0.455)		252 (0.486)		232 (0.441)		162 (0.435)		174 (0.481)	
	336 (0.604)		146 (0.553)		167 (0.596)		195 (0.545)		266 (0.514)		294 (0.559)		210 (0.565)		188 (0.519)	
Control (freq)	270 (0.475)		270 (0.475)		270 (0.475)		270 (0.475)		270 (0.475)		270 (0.475)		270 (0.475)		270 (0.475)	
	298 (0.525)		298 (0.525)		298 (0.525)		298 (0.525)		298 (0.525)		298 (0.525)		298 (0.525)		298 (0.525)	
Odds Ratio (% 95 CI)	0.722663		0.892034		0.746817		0.922583		1.045614		0.870950		0.851429		1.021513	
	(0.570361~0.915634)		(0.665320~1.196004)		(0.558798~0.998101)		(0.707594~1.202893)		(0.823876~1.327031)		(0.686340~1.105216)		(0.654582~1.107471)		(0.784591~1.329979)	
Fisher’s p value	0.0071		0.44502		0.0483		0.55165		0.714		0.255551		0.230431		0.874386	
Genotype	A/A		A/A		A/A		A/A		A/A		A/A		A/A		A/A	
	A/G		A/G		A/G		A/G		A/G		A/G		A/G		A/G	
	G/G		G/G		G/G		G/G		G/G		G/G		G/G		G/G	
Case (freq)	49 (0.176)		23 (0.174)		21 (0.150)		38 (0.212)		51 (0.197)		53 (0.202)		33 (0.177)		41 (0.227)	
	122 (0.439)		72 (0.545)		71 (0.507)		87 (0.486)		150 (0.579)		126 (0.479)		96 (0.516)		92 (0.508)	
	107 (0.385)		37 (0.280)		48 (0.343)		54 (0.302)		58 (0.224)		84 (0.319)		57 (0.306)		48 (0.265)	
Control (freq)	67 (0.236)		67 (0.236)		67 (0.236)		67 (0.236)		67 (0.236)		67 (0.236)		67 (0.236)		67 (0.236)	
	136 (0.479)		136 (0.479)		136 (0.479)		136 (0.479)		136 (0.479)		136 (0.479)		136 (0.479)		136 (0.479)	
	81 (0.285)		81 (0.285)		81 (0.285)		81 (0.285)		81 (0.285)		81 (0.285)		81 (0.285)		81 (0.285)	
Fisher’s p value	0.029		0.30265		0.1048		0.825315		0.063		0.53125		0.317314		0.820064	
HWE for case (df = 1)	0.1697		0.23526		0.527		0.787951		0.01		0.645998		0.497597		0.807632	
HWE for control (df = 1)	0.5011		0.50114		0.5011		0.501122		0.501		0.501136		0.501122		0.501122	

### Expression of CA1 in breast cancer tissues

**Fig. 1:**
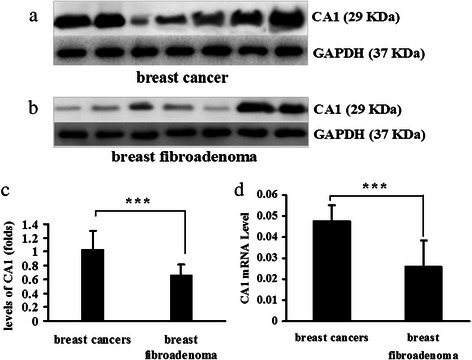
CA1 expression in breast cancer and breast fibroadenoma. **a** Visualisation of CA1 expression and GAPDH expression in breast cancer tissues using western blot analysis. **b** Visualisation of CA1 expression and GAPDH expression in breast fibroadenoma using western blot analysis. **c** For quantification purposes, the level of CA1 protein expression was normalised against GAPDH expression in each of the tissues. **d** Quantifying the expression of CA1 mRNA in breast cancer tissue and breast fibroadenoma using real-time PCR; CA1 expression was normalised against GAPDH expression in each of the tissues. The expression levels are depicted as the means ± SEMs. * indicates *p* < 0.05, ** indicates *p* < 0.01, *** indicates *p* < 0.001

Western blot analysis was performed to detect the expression of CA1 in breast tumour tissue and breast fibroadenoma. Using GAPDH as a reference, western blotting revealed that the expression of CA1 was significantly increased in the breast cancer samples, compared to the breast fibroadenoma samples (*p* = 0.008). Six of seven of the breast cancer samples exhibited increased levels of expression. Only two of seven of the breast fibroadenoma samples exhibited relatively high CA1 expression. These results are shown in Fig. 1.

The transcription of CA1 was also examined using real-time PCR. Similar to the western blotting results above, the breast cancer samples (*n* = 12) exhibited a higher degree of CA1 mRNA expression than the breast fibroadenoma tissue samples (*n* = 12; *p* = 0.0000525). These results are shown in Fig. 1.

**Fig. 2:**
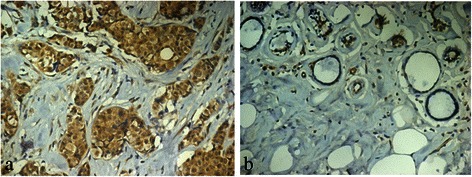
Immunolocalisation of CA1 expression in breast cancer tissues. **a** CA1 expression was significantly increased in breast cancer tissue. **b** CA1 expression was very low in normal breast tissue. The immune signals were located in tumour cells. Original magnification: ×20

Immunohistochemistry was performed to visualise CA1 expression in a panel of breast tumour tissues. There was significant expression of CA1 in 39 of 40 (97.5 %) of the invasive breast ductal carcinomas. There was no obvious expression of CA1 in normal breast tissue except for in select mesenchymal-like cells and select endothelial cells. The immunosignals were localised in the cytoplasm of breast tumour cells. The immunohistochemical results are shown in Fig. 2.

### CA1 levels in blood samples obtained from patients with breast cancer

**Fig. 3:**
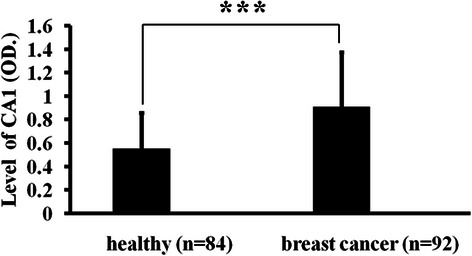
The detection of CA1 levels in patient blood samples using ELISA. The levels of CA1 are represented by O.D. values, which were measured at 405 nm. The results are expressed as the means ± SEMs. * indicates *p* < 0.05, ** indicates *p* < 0.01, *** indicates *p* < 0.001

Sandwich ELISA was used to measure the levels of CA1 in the blood samples that were obtained from patients with breast cancer. There were significantly increased levels of CA1 in the samples that were obtained from breast cancer patients (OD _average_ = 0.91 ± 0.46) compared to the samples that were obtained from healthy controls (OD _average_ = 0.55 ± 0.3). Fourteen of 92 (15.22 %) of the samples that were obtained from patients with breast cancer exhibited two-fold or greater expression of CA1, compared to the average level of expression that was measured in the samples that were obtained from healthy controls. Only one sample from the healthy control group (1.19 %) exhibited a higher level of CA1 expression than the average level in the healthy samples. The serum levels of CA1 were significantly different between breast cancer patients and healthy controls (*p* = 9.18 × 10^−5^). The ELISA results are shown in Fig. 3.

### Bio-mineralisation, cell proliferation and expression of CA1, BSP and OCN in 4T1 cells

**Fig. 4:**
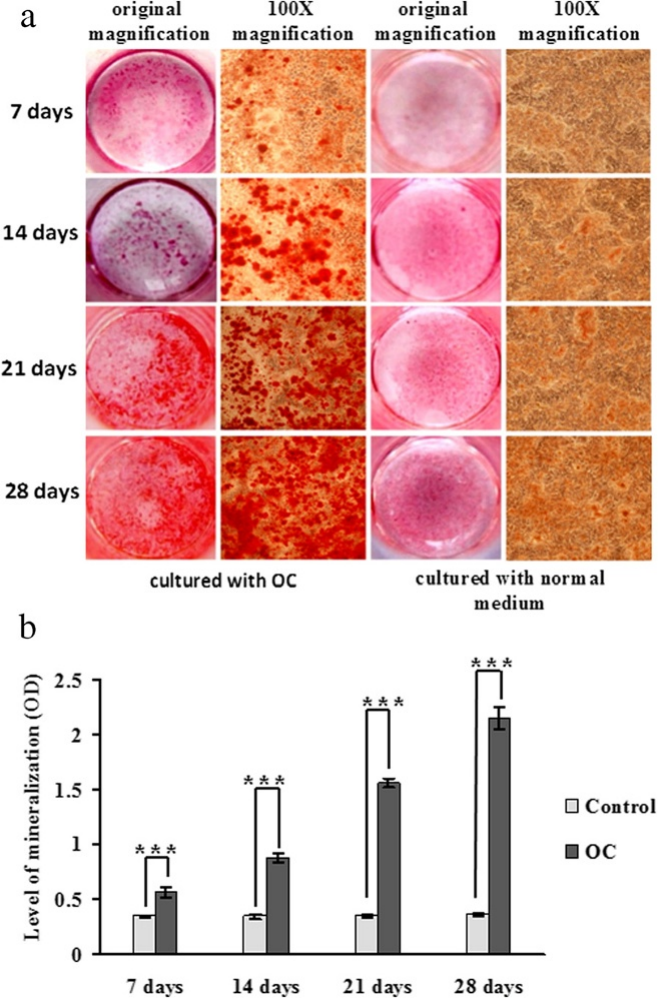
Inducing calcification in 4T1 cells with ascorbic acid and β-glycerophosphate. 4T1 cells were incubated in normal media (control) and an osteogenic cocktail (OC). **a** The cell cultures were stained with AR-S to detect calcium nodules and were photographed at the original magnification and at ×100 magnification. **b** The cell cultures were stained with cetylpyridinium chloride and were quantified by measuring the absorbance at 562 nm. * indicates *p* < 0.05, ** indicates *p* < 0.01, *** indicates *p* < 0.001

Following the induction of bio-mineralisation with OC, numerous calcium deposits were observed in cultured 4T1 cells after incubations of 7, 14, 21 and 28 days. The absorbance of cetylpyridinium chloride was 1.64-fold higher in 4T1 cells that were incubated with OC for 7 days than in cells that were cultured without induction (*p* = 4.32∗10^−6^), and it was 2.57-fold higher at 14 days (*p* = 2.04 × 10^−12^), 4.46-fold higher at 21 days (*p* = 6.05 × 10^−16^), and 6-fold higher at 28 days (*p* = 7.87 × 10^−13^). These results are shown in Fig. 4.

**Fig. 5:**
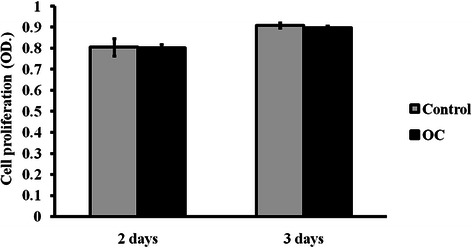
Analysis of cell proliferation using CCK-8 assay. 4T1 cells were incubated in either normal culture media (control) or osteogenic medium (OC). The numbers of viable cells are represented by O.D. values, which were measured at 492 nm. The *error bars* represent the standard deviation from five repeated measurements. * indicates *p* < 0.05, ** indicates *p* < 0.01, *** indicates *p* < 0.001

4T1 cells were incubated with OC for 2 and 3 days, and viable cells were detected using CCK-8 assay. There were no significant differences in the numbers of viable cells that were detected between the 4T1 cells that were cultured in OC and those that were cultured in normal media at either 2 days (*p* = 0.875) or 3 days (*p* = 0.116). These results are shown in Fig. 5.

**Fig. 6:**
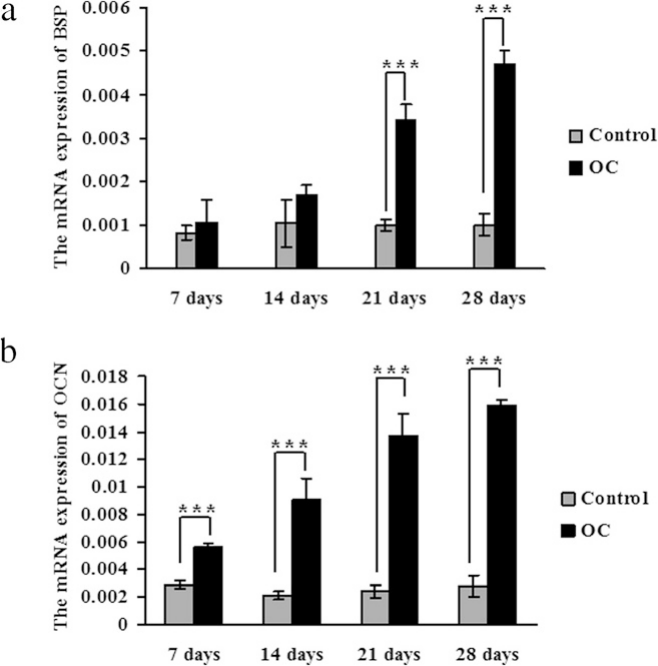
Detecting the mRNA expression of BSP and OCN in 4T1 cells using real-time PCR analysis. 4T1 cells were incubated with an osteogenic cocktail (OC). These cells, which were cultured in normal media, were used as a control. **a** BSP mRNA expression. **b** OCN mRNA expression. The expression was normalised to the expression of GAPDH. * indicates *p* < 0.05, ** indicates *p* < 0.01, *** indicates *p* < 0.001

The level of BSP mRNA was 4.68-fold (*p* = 2.57 × 10^−8^) higher in the OC-stimulated 4T1 cells than in the cells without stimulation, and OCN increased by 5.67-fold (*p* = 3.01 × 10^−5^) when 4T1 cells were incubated in OC for 28 days. These results are shown in Fig. 6. The increased expression of BSP and OCN indicated that the 4T1 cells that were incubated in OC underwent ossification.

**Fig. 7:**
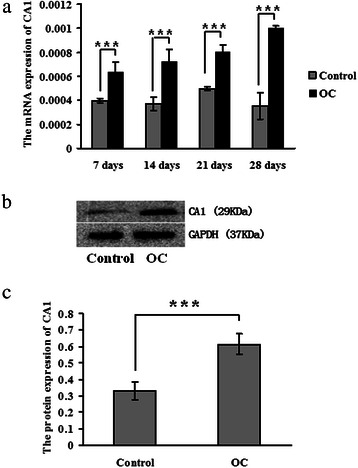
CA1 expression in the calcified 4T1 cells. **a** CA1 transcription was detected using real-time PCR in 4T1 cells that were treated with osteogenic cocktail (OC). **b** CA1 protein expression in 4T1 cells treated with OC was detected using western blot analysis. **c** The protein expression level was normalised to the expression of GAPDH. * indicates *p* < 0.05, ** indicates *p* < 0.01, *** indicates *p* < 0.001

The transcription and translation of CA1 in the OC-stimulated cells were measured using real-time PCR and western blotting, respectively. The level of CA1 mRNA was significantly elevated in the OC-stimulated cells compared with the controls (*p* = 0.00752) after 28 days in culture. The translation of CA1 in the OC-stimulated cells was also significantly increased compared with the controls (*p* = 0.0295). These results are shown in Fig. 7.

**Fig. 8:**
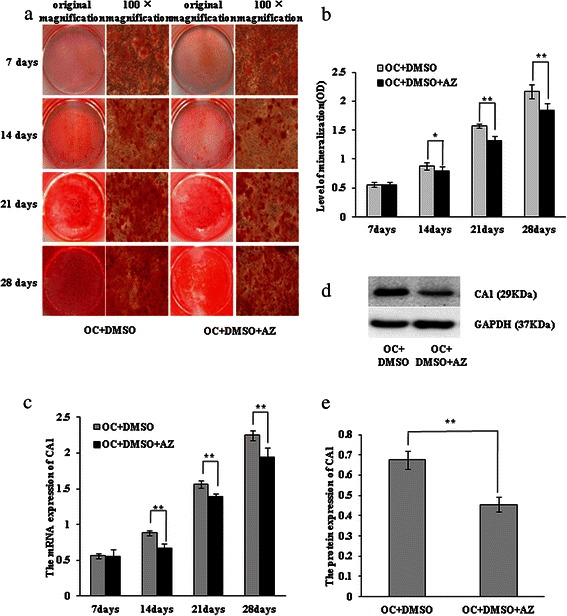
Inhibiting calcification in 4T1 cells with acetazolamide. The 4T1 cells were incubated with osteogenic cocktail (OC) supplemented with 1 mM acetazolamide (AZ). **a** The 4T1 cells cultured with OC supplemented with DMSO in the presence or absence of AZ were stained with AR-S and were photographed at the original magnification and at ×100 magnification. **b** The 4T1 cells, cultured with OC supplemented with DMSO in the presence or absence of AZ, were stained with cetylpyridinium chloride and were quantified by measuring their absorbance at 562 nm. **c** CA1 transcription in 4T1 cells was detected by real-time PCR. **d** CA1 protein expression in 4T1 cells was detected using western blot analysis. **e** The protein expression level was normalised to the expression of GAPDH. * indicates *p* < 0.05, ** indicates *p* < 0.01, *** indicates *p* < 0.001

To identify the essential role of CA1 for calcification in breast cancer cells, acetazolamide, a chemical inhibitor of CA1, was added into the culture medium when 4T1 cells were induced to produce calcification with OC. Compared with 4T1 cells that were treated with OC in the presence of DMSO, the formation of calcium nodules was significantly decreased in the cells cultured with OC in presence of DMSO and acetazolamide for 14, 21 and 28 days. Because acetazolamide was dissolved only in DMSO, the cells cultured with OC supplemented with DMSO were used as a control in the experiment. The absorbance of cetylpyridinium chloride was also considerably decreased in 4T1 cells that were treated with acetazolamide for 14 days (*p* = 0.038), 21 days (*p* = 0.012) and 28 days (*p* = 0.007). This experiment was repeated three times, obtaining similar results. The results are shown in Fig. 8.

### Suppressing CA1 expression in MCF-7 cells using siRNA

**Fig. 9:**
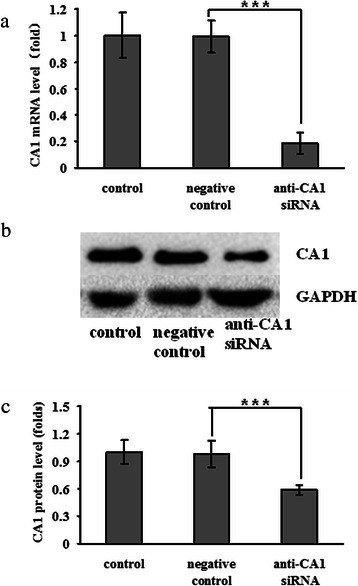
CA1 expression in anti-CA1 siRNA-treated MCF-7 cells. **a** The expression of CA1 mRNA was detected using real-time PCR. **b** The level of CA1 protein expression was detected using western blot analysis. **c** The level of CA1 protein expression was normalised against the expression of GAPDH. The cells without siRNA treatment were used as controls, and the cells treated with Allstars siRNA were used as negative controls. * indicates *p* < 0.05, ** indicates *p* < 0.01, *** indicates *p* < 0.001

MCF-7 cells were transfected with siRNA targeted to CA1. The transcription and translation of CA1 in the treated cells were measured using real-time PCR and western blotting, respectively. Following the anti-CA1 siRNA treatment, both the transcription (*p* = 0.004) and the translation (*p* = 0.0003) of CA1 were considerably reduced in the siRNA-treated MCF-7 cells, compared with the cells that were treated with the Allstars siRNA negative control and the cells that did not receive siRNA treatment. Neither the control nor negative control siRNA led to a significant change in the expression of CA1 mRNA, indicating the specificity of the RNA interference with regard to CA1 expression in this experiment. These results are shown in Fig. 9.

**Fig. 10:**
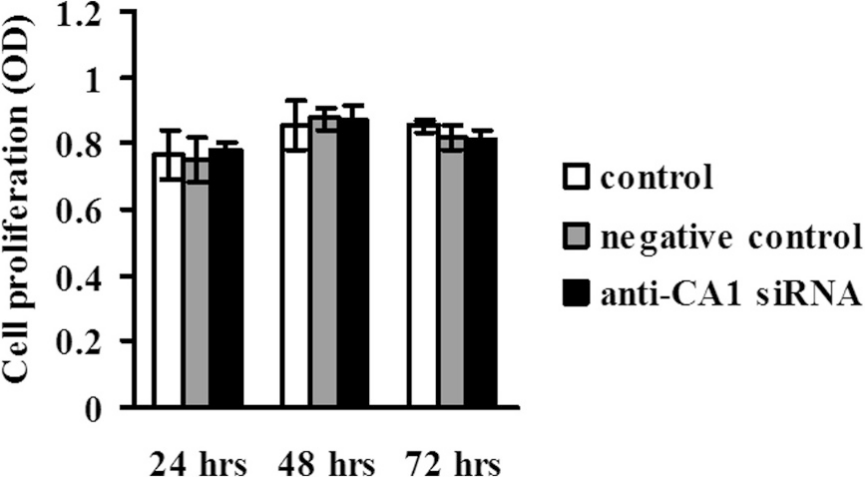
Proliferation of MCF-7 cells that were treated with anti-CA1 siRNA, as measured by CCK-8 assay. Viable cell numbers are represented by an O.D. value that was measured at 405 nm. The cells without siRNA treatment were used as controls, and the cells treated with Allstars siRNA were used as negative controls. *indicates *p* < 0.05, **indicates *p* < 0.01, ***indicates *p* < 0.001

The proliferation of MCF-7 cells that were treated with anti-CA1 siRNA was measured using CCK-8 assay. No significant change in cell proliferation was detected in the siRNA-treated MCF-7 cells, compared to the cells that were treated with the negative siRNA control (*p* = 0.981) and the cells that were treated only with HiPerFect transfection reagent (*p* = 0.801). These results, which are shown in Fig. 10, indicated that CA1 did not affect MCF-7 cell proliferation.

**Fig. 11:**
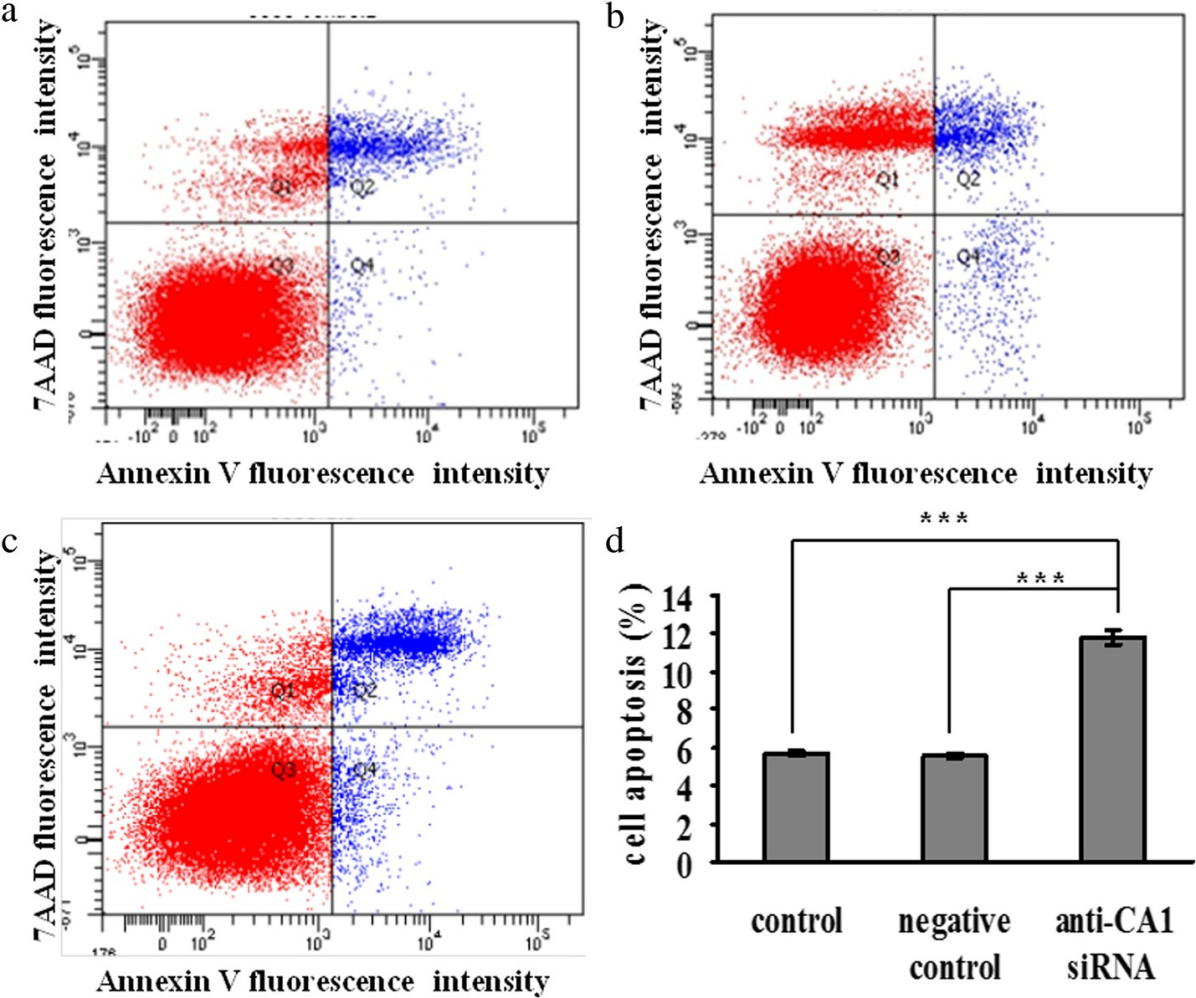
Apoptosis in MCF-7 cells that were treated with anti-CA1 siRNA, as measured using an annexin V cell apoptosis assay. (**a**) The cells without siRNA treatment were used as controls. (**b**) The cells treated with Allstars siRNA were used as negative controls. (**c**) The cells were treated with anti-CA1 siRNA. (**d**) The result of apoptosis assay is shown in a graph. *indicates p < 0.05, **indicates p < 0.01, ***indicates p < 0.001.

The effect of CA1 on apoptosis in MCF-7 cells was determined using flow cytometric analysis with annexin V/PE and 7-AAD double staining. Compared with the negative control (5.57 ± 0.15 %), the number of apoptotic cells was significantly increased in the anti-CA1 siRNA-treated cells (*p* = 9.14E-8). No significant differences were detected between the control and negative control groups (*p* = 0.547). These results, shown in Fig. 11, demonstrate that down-regulation of CA1 expression could induce apoptosis in MCF-7 cells.

**Fig. 12:**
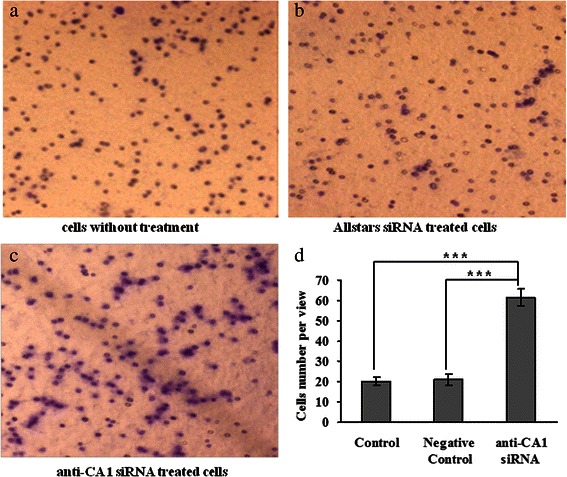
Migration of MCF-7 cells that were treated with anti-CA1 siRNA, as measured using a transwell migration assay. (**a**) The cells without siRNA treatment were used as controls. (**b**) The cells treated with Allstars siRNA were used as negative controls. (**c**) The cells were treated with anti-CA1 siRNA. (**d**) The result of migration measurement is shown in a graph. Original magnification: x4.2. * indicates p < 0.05, **indicates p < 0.01, ***indicates p < 0.001.

MCF-7 cell migration was examined using a 2-compartment transwell system. MCF-7 cell migration increased significantly when CA1 expression was suppressed by anti-CA1 siRNA (*p* < 0.001). No significant difference in migration was observed between the control cells and the cells that were treated with negative control siRNA (*p* > 0.05). These results are shown in Fig. 12.

**Fig. 13:**
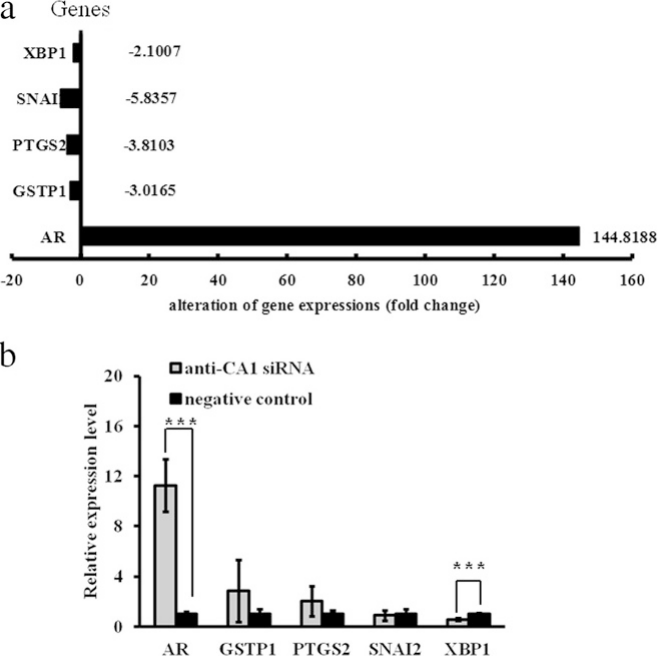
Human Breast Cancer RT^2^ Profiler™ PCR array analysis of signalling pathways in anti-CA1 siRNA-treated MCF-7 cells. **a** The expression levels of four genes, including GSTP1, PTGS2, SNAI2 and XBP1, were down-regulated, whereas the expression of AR was up-regulated in the anti-CA1 siRNA-treated cells. **b** Real-time PCR analysis of AR, GSTP1, PTGS2, SNAI2 and XBP1 expression in MCF-7 cells that were treated with anti-CA1 siRNA. The transcription levels of the target genes were normalised to their corresponding expression levels in the cells that were treated with Allstars siRNA. Error bars indicate the standard error of the mean. * indicates *p* < 0.05, ** indicates *p* < 0.01, *** indicates *p* < 0.001

To identify the details of CA1’s involvement in breast cancer tumourigenesis, a PCR array containing 84 different breast cancer-related genes was used to compare the expression profiles of MCF-7 cells that exhibited down-regulated CA1 expression and MCF-7 cells that were treated with AllStars siRNA. Five different breast cancer-related genes exhibited significantly different expression patterns (4-fold or greater changes) in anti-CA1 siRNA-treated MCF-7 cells. The expression of AR (androgen receptor) gene expression was up-regulated, whereas the expression of the GSTP1 (glutathione S-transferase pi 1), PTGS2 (prostaglandin-endoperoxide synthase 2), SNAI2 (snail family zinc finger 2) and XBP1 (X-box binding protein 1) genes was down-regulated in the cells. These results are shown in Additional file 1 and Fig. 13a.

The results of the PCR array were verified with real-time PCR. Compared with the AllStars siRNA-treated MCF-7 cells, the average expression level of AR was significantly elevated (*p* < 0.001), and the average expression level of XBP1 was significantly reduced (*p* < 0.01) in the anti-CA1 siRNA-treated MCF-7 cells. No significantly different expression patterns in the GSTP1, PTGS2, or SNAI2 genes were detected in the CA1-siRNA cells. The real-time PCR results are shown in Fig. 13b.

## Discussion

Despite the diagnostic and potential prognostic value of microcalcification, the mechanisms underlying the formation and functional role of microcalcification in breast cancer progression remain unclear. In the present study, we genotyped Tag SNP rs725605 in cohorts of patients with various types of tumours and found that this tag SNP, which is located in the intron 1 region of the gene that encodes CA1, was strongly associated with breast cancer but with no other tumours. The results suggested that CA1 is a risk factor for breast cancer. Additionally, increases in CA1 transcription and translation were detected in breast cancer tissue compared to breast fibroadenoma tissue via western blot and RT-PCR analysis. Benign breast lesions exhibit calcification similar to breast cancer tissues. Thus, it was feasible in this study to use fibroadenoma breast tissue as a control when studying calcification in breast tumours [20]. Using immunohistochemistry, extensive expression of CA1 was detected in breast cancer tissues but not in normal tissues. Furthermore, ELISA indicated that the levels of CA1 were significantly increased in several of the blood samples that were obtained from patients with breast cancer. These results suggested that increased CA1 expression might play a role in breast cancer tumourigenesis.

In the current study, we aimed to identify the role of CA1 in mammary calcification by inducing mineralisation of 4T1 cells. Following stimulation with ascorbic acid and β-glycerophosphate, numerous calcium-rich deposits were produced in 4T1 cells, and the transcription levels of osteogenesis-related genes, including BSP and OCN, were significantly increased, indicating that the cells underwent calcification and ossification. CA1 expression was significantly increased in the OC-stimulated cells during calcification. In addition, the OC did not significantly increase cell proliferation. Thus, the observed increase in mineralisation was due to a direct effect on mineralisation, rather than because of increased cell numbers. The expression levels of CA1 and calcification were significantly decreased when the cells were incubated with acetazolamide, a chemical inhibitor of CA1. These results suggested that increased expression of CA1 promotes the calcification of breast cancer. We knocked down CA1 expression in MCF-7 cells using siRNA. The down-regulation of CA1 expression significantly stimulated cell apoptosis and cellular migration but did not influence cell proliferation, consistent with what was observed in 4T1 cells that were subjected to an induction of calcification. These results suggested that CA1 expression also mediates apoptosis and cell migration, which are two important steps in the process of tumourigenesis.

The pathogenic pathway of CA1 in breast cancer tumourigenesis was investigated using a PCR array that contained 84 different breast cancer-related genes. The results were verified with real-time PCR. The assay detected increased expression of the AR gene and decreased expression of the XBP1 gene in MCF-7 cells following anti-CA1 siRNA treatment, suggesting that CA1 functions by regulating the expression of AR and XBP1.

AR expression has been reported in approximately 80 % of primary breast cancers [21]. Hu et al. reported that a significant reduction in breast cancer mortality was associated with AR expression in patients with ER (oestrogen receptor) + cancers [22]. Other groups have also reported that AR expression was associated with favourable outcomes in patients with ER+ breast cancers [22-24]. Yeh et al. suggested that AR might play a positive role in promoting breast cancer progression [25]. Additionally, Venken et al. demonstrated that AR-knockout mice exhibited reduced bone size and cortical thickness and decreased trabecular bone volume [26]. Androgen/AR signalling plays an essential role in bone formation by coordinating the expression of genes that are associated with phosphate regulation [25-27]. Our results revealed that inhibiting CA1 expression could increase the expression of AR, suggesting that CA1 could regulate AR expression and might therefore influence the progression of microcalcification and other tumourigenic processes during breast cancer tumourigenesis.

XBP1 is a transcription factor that belongs to the basic region/leucine zipper (bZIP) family [28]. The XBP1 protein is expressed in almost 80 % of ER+ breast tumours. The overexpression and splicing of XBP1 have been associated with poor outcomes in breast cancer patients [29]. XBP1 is downstream of NFκB, which regulates cell survival via activation of the anti-apoptotic BCL2 (B-cell lymphoma 2) gene, as well as several other genes that are associated with control of the cell cycle and apoptosis [30]. Additionally, XBP1 signalling increases the expression of Runx2, which modulates calcification in human coronary artery smooth muscle cells [31]. The present study found that decreased expression of CA1 led to decreased expression of XBP1, consistent with previous studies. This finding suggested that CA1 enhances XBP1 gene expression, thereby contributing to calcification and disrupted apoptosis in breast cancer cells.

Our study indicated that the down-regulation of CA1 expression significantly stimulated cell apoptosis, but it did not influence cell proliferation in MCF-7 cells. We also found that the expression of XBP1 was decreased in the anti-CA1 siRNA-treated MCF-7 cells. XBP1 can activate the anti-apoptotic BCL2 gene [30]. Thus, it is possible that the decreased CA1 expression in the cultured breast cancer cells suppressed the anti-apoptotic role of BCL2, resulting in increased levels of apoptosis in the treated cells. Apoptosis is not always correlated with cell proliferation. For example, Gálfi et al. investigated a set of cell lines to assess their sensitivity to proliferation inhibition in response to the core histone deacetylase inhibitors butyrate and trichostatin A. They found that there was no correlation between sensitivity to proliferation inhibition and sensitivity to apoptosis induction with respect to the histone deacetylase inhibitors [32].

Increased migration and apoptosis were observed in the anti-CA1 siRNA-treated MCF-7 cells, as well as an increased expression of AR. Numerous studies have reported that AR stimulated tumour cell migration, such as during prostate cancer cell invasion and oesophageal cancer cell migration [33,34]. Apoptosis is disrupted during the progression of many types of solid tumours. In contrast, apoptosis is not always negatively correlated with tumourigenic progression, especially in cases of metastasis. For example, Termuhlen et al. reported that isogenic metastatic colon adenocarcinoma cells displayed significantly higher levels of staurosporine-induced apoptosis than their nonmetastatic counterparts in vitro [35]. They suggested that the molecular events that were associated with the development of a metastatic phenotype sensitised the tumour cells to selecting pro-apoptotic stimuli.

In the present study, we induced calcification in the 4T1 cell line to determine whether a correlation existed between CA1 and bio-mineralisation because Cox et al. established an excellent *in vitro* model of mammary mineralisation using this cell line. However, 4T1 is mouse cell line; therefore, it might not be physiologically relevant to human breast cancer. Thus, we also investigated CA1 transcription and translation in MCF-7 cells, which originated from human breast tumour epithelium. MCF-7 cells have been reported to express alkaline phosphatase and bone sialoprotein, both of which enhance bone mineralisation and calcification [36, 37]. Thus, MCF-7 cells could be induced to produce calcification. Carbonic anhydrases catalyse the rapid inter-conversion of carbon dioxide and water into bicarbonate and protons, respectively, which can affect physiological processes in cells by altering pH, proton concentrations, and membrane permeability. In addition, calcium hydroxyapatite has been reported to promote mitogenesis and matrix metalloproteinase expression in varying human breast cancer cell lines [38].

## Conclusion

This study demonstrated that CA1 expression was increased in samples of breast cancer tissues and blood obtained from patients with breast cancer. CA1 plays important roles in the calcification, apoptosis and migration of tumour cells and contributes to breast cancer tumourigenesis by regulating the expression of AR and XBP1.

## Additional file

Additional file 1: **Human Breast Cancer RT**^**2**^ **Profiler™ PCR array analysis of gene expression patterns in anti-CA1 siRNA-treated MCF-7 cells.** (a) The expression patterns of 84 different genes related to breast cancer tumourigenesis were measured. Fold-change values greater than one indicated a positive finding or up-regulation. Fold-change values less than one indicated a negative finding or down-regulation. Fold-change values greater than 2 are indicated in red; fold-change values less than 0.5 are indicated in blue. (b) A table indicates expression levels of the 84 different genes. Comments with “A” indicate that the average threshold cycle of the gene expression is relatively high (>30) in either the control or the test sample and is reasonably low in the other sample (< 30). These data indicate that the gene expression is relatively low in one sample and reasonably detected in the other sample, suggesting that the actual fold-change value is at least as large as the calculated and reported fold-change results. This fold-change result might also have greater variations if the *p* value > 0.05. Comments with “B” indicate that the average threshold cycle of the gene expression is relatively high (> 30), indicating that its relative expression level is low in both control and test samples, and the p value for the fold-change is either unavailable or relatively high (*p* > 0.05). This fold-change result might also have greater variations. Comment with “C” indicates that the average threshold cycle of the gene expression is either not determined or greater than the defined cut-off value (default 35) in both samples, indicating that its expression was undetected, thus rendering this fold-change result erroneous and un-interpretable.
