# Supplementary material for: CA1 contributes to microcalcification and tumourigenesis in breast cancer
Source: BMC Cancer. 2015 Oct 12;15:679. doi: 10.1186/s12885-015-1707-x (PMC4603971; doi:10.1186/s12885-015-1707-x)
Supplement: Additional file 1: — Human Breast Cancer RT2 Profiler™ PCR array analysis of gene expression patterns in anti-CA1 siRNA-treated MCF-7 cells. (a) The expression patterns of 84 different genes related to breast cancer tumourigenesis were measured. Fold-change values greater than one indicated a positive finding or up-regulation. Fold-change values less than one indicated a negative finding or down-regulation. Fold-change values greater than 2 are indicated in red; fold-change values less than 0.5 are indicated in blue. (b) A table indicates expression levels of the 84 different genes. Comments with “A” indicate that the average threshold cycle of the gene expression is relatively high (>30) in either the control or the test sample and is reasonably low in the other sample (< 30). These data indicate that the gene expression is relatively low in one sample and reasonably detected in the other sample, suggesting that the actual fold-change value is at least as large as the calculated and reported fold-change results. This fold-change result might also have greater variations if the p value > 0.05. Comments with “B” indicate that the average threshold cycle of the gene expression is relatively high (> 30), indicating that its relative expression level is low in both control and test samples, and the p value for the fold-change is either unavailable or relatively high (p > 0.05). This fold-change result might also have greater variations. Comment with “C” indicates that the average threshold cycle of the gene expression is either not determined or greater than the defined cut-off value (default 35) in both samples, indicating that its expression was undetected, thus rendering this fold-change result erroneous and un-interpretable. [file 12885_2015_1707_MOESM1_ESM.docx]

**
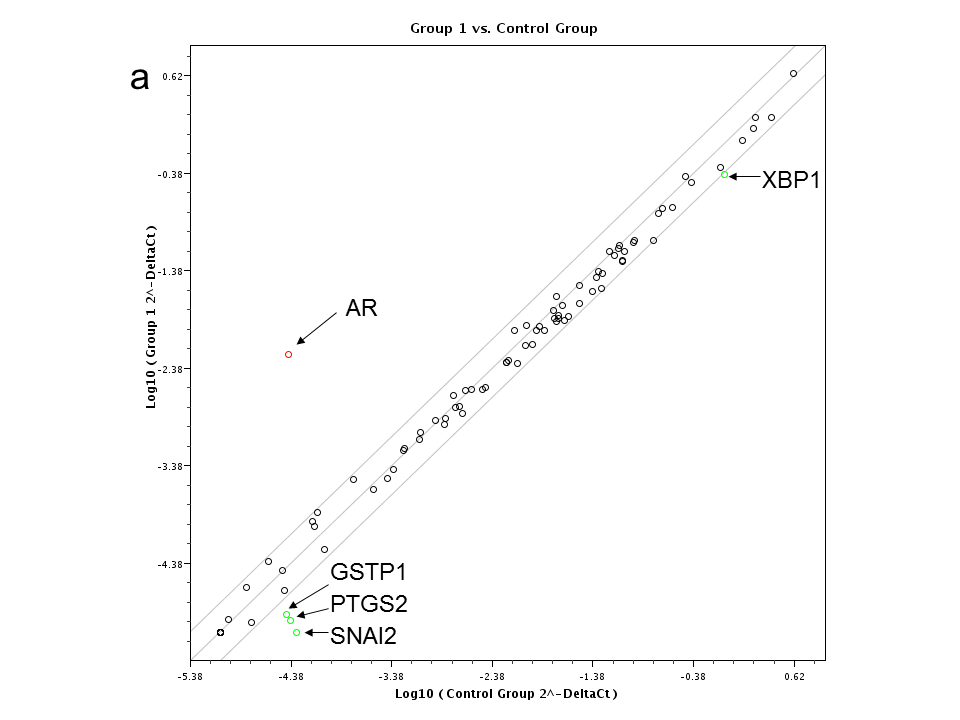
**

**Additional file 1b. Human Breast Cancer RT² Profiler™ PCR array analysis of gene expression patterns in anti-CA1 siRNA-treated MCF-7 cells.**

|  | Layout | 1 | 2 | 3 | 4 | 5 | 6 | 7 | 8 | 9 | 10 | 11 | 12 |
| --- | --- | --- | --- | --- | --- | --- | --- | --- | --- | --- | --- | --- | --- |
| A | Genes | ABCB1 | ABCG2 | ADAM23 | AKT1 | APC | AR | ATM | BAD | BCL2 | BIRC5 | BRCA1 | BRCA2 |
|  | fold-change | 1.77 | -1.09 | -1.58 | -1.29 | 1 | 144.82 | 1.11 | -1.11 | -1.48 | -1.14 | -1.61 | -1.31 |
|  | comments | B | OKAY | B | OKAY | OKAY | A | OKAY | OKAY | OKAY | OKAY | OKAY | OKAY |
| B | Genes | CCNA1 | CCND1 | CCND2 | CCNE1 | CDH1 | CDH13 | CDK2 | CDKN1A | CDKN1C | CDKN2A | CSF1 | CST6 |
|  | fold-change | -1.23 | -1.97 | 1.58 | -1.21 | -1.15 | -1.02 | -1.67 | -1.08 | 1.28 | -1.02 | -1.12 | -1.02 |
|  | comments | OKAY | OKAY | B | OKAY | OKAY | C | OKAY | OKAY | OKAY | C | OKAY | C |
| C | Genes | CTNNB1 | CTSD | EGF | EGFR | ERBB2 | ESR1 | ESR2 | FOXA1 | GATA3 | GLI1 | GRB7 | GSTP1 |
|  | fold-change | -1.59 | -1.19 | 1.37 | -1.29 | -1.19 | -1.68 | -1.66 | -1.26 | -1.01 | -1.67 | 1.09 | -3.02 |
|  | comments | OKAY | OKAY | B | OKAY | OKAY | OKAY | B | OKAY | OKAY | B | OKAY | B |
| D | Genes | HIC1 | ID1 | IGF1 | IGF1R | IGFBP3 | IL6 | JUN | KRT18 | KRT19 | KRT5 | KRT8 | MAPK1 |
|  | fold-change | 1.04 | -1.28 | -1.02 | -1.57 | 1.47 | 1.12 | -1.25 | -1.11 | -1.42 | -1.02 | 1.11 | -1.14 |
|  | comments | B | OKAY | C | OKAY | OKAY | B | OKAY | OKAY | OKAY | C | OKAY | OKAY |
| E | Genes | MAPK3 | MAPK8 | MGMT | MKI67 | MLH1 | MMP2 | MMP9 | MUC1 | MYC | NME1 | NOTCH1 | NR3C1 |
|  | fold-change | -1.14 | -1.41 | -1.04 | -1.64 | -1.41 | -1.02 | -1.02 | 1.07 | -1.57 | -1.37 | -1.13 | 1.26 |
|  | comments | OKAY | OKAY | OKAY | OKAY | OKAY | C | OKAY | OKAY | OKAY | OKAY | OKAY | OKAY |
| F | Genes | PGR | PLAU | PRDM2 | PTEN | PTGS2 | PYCARD | RARB | RASSF1 | RB1 | SERPINE1 | SFN | SFRP1 |
|  | fold-change | -1.37 | 1.8 | -1.37 | -1.04 | -3.81 | -1.14 | 1.74 | 1.07 | -1.3 | 1.64 | -1.11 | -1.02 |
|  | comments | OKAY | B | OKAY | OKAY | B | OKAY | A | OKAY | OKAY | B | OKAY | C |
| G | Genes | SLC39A6 | SLIT2 | SNAI2 | SRC | TFF3 | TGFB1 | THBS1 | TP53 | TP73 | TWIST1 | VEGFA | XBP1 |
|  | fold-change | -1.62 | 1.11 | -5.84 | -1.22 | 1.26 | -1.31 | -1.85 | -1.29 | -1.15 | 1.08 | -1.42 | -2.1 |
|  | comments | OKAY | OKAY | B | OKAY | OKAY | OKAY | OKAY | OKAY | A | OKAY | OKAY | OKAY |
